# Ultrasound assisted homing of human umbilical cord mesenchymal stem cells promotes recovery from acute respiratory distress syndrome

**DOI:** 10.1186/s13287-025-04545-6

**Published:** 2025-07-26

**Authors:** Bei-Ying Wang, Xiao Zhang, Ting-Tian Li, Wei-Wei Qin, Xiang Liu, Kong-Miao Lu, Li-Xin Sun, Wei Han

**Affiliations:** 1 Department of Respiratory and Critical Care Medicine, Qingdao Municipal Hospital, Nanjing Medical University, Nanjing, 211166 China; 2 Department of Emergency and Critical Care, Qingdao Municipal Hospital, University of Health and Rehabilitation Sciences, Qingdao, 266071 China; 3 Department of Anesthesiology, Qingdao Municipal Hospital, Qingdao, 266071 China

**Keywords:** Human umbilical cord mesenchymal stem cells, Ultrasound, Acute respiratory distress syndrome

## Abstract

**Background:**

Human umbilical cord mesenchymal stem cells (hUC-MSCs) show potential for treating acute respiratory distress syndrome (ARDS), however, their homing to the lungs and survival time are insufficient. In this study, we evaluated whether pulsed focus ultrasound (pFUS) could promote the homing and prolonged retention of hUC-MSCs in the lungs of ARDS mice and explored the mechanisms involved.

**Methods:**

Mice were divided into four groups: the NC group, the LPS group, the MSCs group, and the pFUS + MSCs group. Except for the NC group, the other three groups were constructed as ARDS models and given PBS, MSCs and pFUS + MSCs interventions. hUC-MSCs were used to assess lung tissue injury by HE staining, inflammatory cell count in alveolar lavage fluid (BALF), and expression of *Tnf*, *Il1b and Il6* in the lung tissues; and apoptosis and proliferation in the lung tissues were assessed by TUNEL and immunofluorescence. Bioluminescence imaging was used to detect the homing rate and survival of hUC-MSCs in mouse lungs from 1 to 7 days. *Cxcl5 and Igf1* was found to be differentially expressed and highly enriched by mRNA sequencing in MSC and sonicated groups and verified by PCR combined with ELISA.

**Results:**

Compared with the LPS group, the lung inflammatory infiltrate and lung tissue damage in the MSCs group and pFUS + MSC group were alleviated, the number of inflammatory cells in the BALF and the expression of *Tnf*, *Il1b and Il6* in the lung tissues were reduced, the expression of TUNEL-positive cells was reduced, and the expression of PCNA-positive cells was increased, and the decrease or increase was more significant in the pFUS + MSC group (*P* < 0.05). pFUS increased the number of hUC-MSCs homing in the lungs and prolonged lung survival to day 6 and significantly up-regulated lung tissue levels of SDF-1, ICAM-1, CXCL5 and IGF-1 compared to the MSCs group (*P* < 0.05).

**Conclusions:**

pFUS preconditioning may improve lung homing and prolong survival of hUC-MSCs by upregulating the levels of homing-associated factors SDF-1, ICAM-1, CXCL5 and IGF-1, which in turn improves ARDS.

**Supplementary Information:**

The online version contains supplementary material available at 10.1186/s13287-025-04545-6.

## Introduction

Acute Respiratory Distress Syndrome (ARDS) is a severe respiratory condition characterized by noncardiogenic bilateral pulmonary edema and profound hypoxemia. It arises from damage to the alveolar epithelial-endothelial barrier, resulting in increased pulmonary capillary permeability [1, 2]. Patients with ARDS often suffer from significant cellular dysfunction, inflammation, immune dysregulation, and oxidative stress, placing them at high risk for pulmonary fibrosis and multiorgan failure. Current treatments remain largely inadequate, underscoring the urgent need for innovative therapeutic strategies to improve patient outcomes [3].

Mesenchymal stem cells (MSCs), an emerging therapeutic modality, are now emerging as a powerful tool in the treatment of many diseases previously considered incurable [4]. The specificity of stem cell therapy lies in its strong immunomodulatory properties, pro-angiogenicity, reconstructive effect on tissue structure and correction of inflammatory imbalances in the body, so it plays a therapeutic role in many acute and critical diseases, including ARDS, and is a very promising approach [5, 6]. It has been discovered that the homing of MSCs is a crucial aspect of their functionality, which is divided into five distinct steps, (1) tethering, (2) activation, (3) adhesion, (4) transmigration or diapedesis, and (5) migration, in which a variety of chemokines and adhesion factors, such as stromal cell-derived factor (SDF-1) and intercellular cell adhesion molecule (ICAM-1) are respectively involved in activation and adhesion phases of MSCs homing [7, 8]. However, the ability of MSCs to home to the target damaged tissues is insufficient and the survival time is relatively short, the majority of MSCs tend to die within a short period, which in turn significantly impacts the effectiveness of the treatment. [9, 10]. Therefore, enhancing the homing efficiency of MSCs to damaged areas has emerged as a critical priority in the field of regenerative medicine.

Previous research has identified a range of strategies designed to enhance the homing of MSCs to specific target tissues, these include magnetic guidance, genetic modification, and cell surface engineering, among others, however, these approaches are often either invasive to the organism or overly complex and challenging to implement [7, 11]. Pulsed focused ultrasound (pFUS), which exerts mechanical forces of acoustic radiation forces (ARF) and cavitation on tissues, generates intracellular Ca2 + transients in several cell types by activating mechanosensitive ion channels[12]. It can promote a transient increase in cytokines, chemokines and trophic factors at the site of local irradiation, which in turn affects the activation, adhesion and migration steps of MSC homing. By enhancing these key steps, pFUS promotes the homing of MSCs to the target tissue without causing tissue damage [7, 12, 13]. However, it is unclear whether pFUS can efficiently facilitate the homing and retention of hUC-MSCs in the lungs of ARDS mice.

Consequently, the objective of our current investigation was to assess the impact of pFUS on directing human umbilical cord mesenchymal stem cells (hUC-MSCs) to injured lung tissues. Moreover, our study sought to elucidate the underlying mechanisms through which pFUS enhances cell engraftment and its potential therapeutic effects on ARDS.

## Materials and methods

### Cultivation, characterization and transduction of hUC-MSCs

The hUC-MSCs used in this experiment were supplied by Qingdao Zhong De Hua Da Cell Technology Co, MSC were cultured using Human Umbilical Cord MSC Serum Free Medium (Applied Cell) in T175 culture flasks at 37 °C in an incubator with 5% CO_2_ concentration. The cells of doubling time approx. 22 were used in this experimental study for cycle and phenotypic characterisation. The hUC-MSCs induced differentiation experiments were performed using alizarin red staining to verify osteogenesis, oil red O staining to verify lipogenesis, and alizarin blue staining to verify chondrogenesis. The cycle of hUC-MSCs was detected by Cell Cycle and Apoptosis Analysis Kit (Beyotime). Relevant cell surface markers such as CD73, CD90, CD105, CD34, CD45, CD11b and HLA-DR were detected by flow cytometry. Cells were transfected using lentivirus expressing enhanced green fluorescent protein (EGFP) for transduction (Genechem), the required viral volume was calculated as:Viral volume = (number of cells × MOI)/viral titer.

### Research design of animal experiments

Male C57BL/6 mice at 6–8 weeks of age were provided by Jinan Pengyue Laboratory Animal Breeding Co., Ltd. This experiment was approved by the Animal Ethics Committee of Qingdao Municipal Hospital. In the study, ARDS was induced in mice using a 5 mg/kg LPS airway drip (Sigma), while the control group received saline [14]. To select the optimal pFUS parameters, 16 ARDS mice were randomly divided into 4 groups (1) Control group (untreated ARDS mice), (2) 1.0 W/cm^2^ group, (3) 1.5 W/cm^2^ group, and (4) 2.0 W/cm^2^ group. Mice were treated with ultrasonic irradiation 6 h after airway drip LPS, a therapeutic ultrasound (Sonopuls190, XIBOY) was used in this experiment. Anesthetized mice were shaved at the chest area with electric clippers and placed horizontally on a stationary operating table. The ultrasound probe (area 0.8 cm^2^) was placed above the mouse chest and covered with a layer of gel. The other acoustic parameters were as follows: 1 MHz, 20% duty cycle, and exposure time of 5 min [15]. Mice were anaesthetised and euthanised with sodium pentobarbital after 48 h. Lung tissues were taken for histopathological examination and the expression of SDF-1, tumour necrosis factor α (*Tnf*) and *Il1b*.

After selecting the pFUS parameters, 30 mice were divided into four groups: (1) NC (6 mice), (2) LPS (untreated ARDS mice: 6 mice), (3) MSCs transplantation (hUC-MSCs + LPS: 9 mice) and (4) treatment with pFUS + MSCs (pFUS + hUC-MSCs + LPS: 9 mice). The NC group was titrated with an equal amount of saline, and the pFUS + MSCs group underwent chest ultrasound irradiation, and the MSCs group and pFUS + MSCs group were immediately injected slowly with 1 × 10^6^/200 μL hUC-MSCs via the tail vein [14]. The LPS group was injected 200 μL PBS. Forty-eight hours after injection of hUC-MSCs, all animals were euthanised using pentobarbital solution (100–200 mg/kg) to collect bronchoalveolar lavage fluid (BALF) and lung tissue. The work has been reported in line with the ARRIVE guidelines 2.0.

### Bioluminescence imaging

To examine the homing rate and retention of hUC-MSCs in the lungs of mice from days 1–7, cells were transfected with EGFP- and luciferase-labeled lentiviral vectors, mice in the MSCs group and the pFUS + MSCs group were imaged bioluminescently from day 1–7 after injection of lentivirus-labelled hUC-MSCs into the tail vein, and each mouse was injected intraperitoneally with 10 μg/g of D-luciferin sodium salt (yeasen, China) at a concentration of 15 mg/mL prior to imaging, and imaged in a bioluminescence imager after 15 min.

### Analysis of total protein, cell volume and inflammatory cytokines in BALF

BALF from the right lung of mice was collected and placed in a freezers at -80 °C. The total protein in the BALF was detected using the BCA Protein Assay Kit. TNF-α, insulin-like growth factor 1 (IGF-1) and chemokine CXCL5 were detected using ELISA (Elabscience). BALF was stained using Giemsa (B.Y.T) and neutrophil and macrophage counts were performed under a light microscope.

### Pulmonary histopathology

After collection of BALF, left lung tissue was fixed in 4% paraformaldehyde and embedded in paraffin for fixation. Then, paraffin-embedded Sects. (4 μm) were stained with hematoxylin–eosin (HE) to assess the level of lung inflammation and lung tissue damage scores[16].

### RNA sequencing and bioinformatic analysis

Total RNA was extracted from mouse lung tissue using the TRIzol method. rRNA was removed after measuring the concentration and purity of the RNA to generate a sequencing library. The first-strand cDNA was synthesised using random hexamer primers and reverse transcriptase, and the quality of the library was assessed, and the average insertion fragment of the cDNA library was determined to be 150 ~ 200 bp.

After clustering was generated, the transcriptome was assembled using StringTie. FPKM is the gene expression level estimated based on the number of fragments per kilobase on the transcript fragment map. mRNA FPKM was estimated based on the number of fragments per kilobase on the transcript fragment map. mRNA FPKM was calculated using StringTie (1.3.1), and differentially expressed mRNAs with FC (fold-change) > 1.5 and FDR value < 0.05 were identified using the DESeq2 package Finally, the constructed libraries were analysed by online sequencing using illumina novaseq6000 (Shandong Biomarker Technology Co., Ltd.). The original data has been uploaded to the NCBI database (PRJNA1228302).

### Western blotting

Two chemokines involved in the migration of hUC-MSCs, SDF-1 and intercellular cell adhesion molecule (ICAM-1) were examined for their expression in the lungs. Protein equal amount of lung tissue was taken and configured with the appropriate amount of protein lysate as well as protease inhibitor according to the instructions, the supernatant was collected, and about 40 μg of protein was taken from each sample and separated on 8%, 10%, and 12% SDS–polyacrylamide gels, respectively. Proteins were electrophoretically transferred to PVDF membranes by wet transfer, closed for 5 min at room temperature using a protein-free rapid closure solution (Servicebio), and incubated overnight with the following antibodies: anti-SDF-1(1:1000, Abcam), anti-ICAM-1(1:2000, Proteintech), the membrane was exposed to horseradish peroxidase (HRP)-coupled goat anti-rabbit/anti-mouse IgG secondary antibody (1:10,000, Proteintech) for 1 h at room temperature. Finally, target bands were visualized using enhanced chemiluminescence reagents (Millipore), quantified using ImageJ software, and normalized according to the internal control GAPDH.

### qRT-PCR

To verify the changes in cytokine expression levels, mRNA expression of *Sdf1*, *Icam1*, *Tnf*, *Il6*, *Il1b*, *Igf1 and Cxcl5* was detected using qRT-PCR. Total RNA was extracted from frozen lung tissues using Trizol reagent (Invitrogen) according to the manufacturer's instructions, reverse transcribed, and then subjected to RT-PCR (Takara) using the appropriate kit according to the manufacturer's instructions The forward and reverse primers for the target genes are listed in Table 1 (generated by Sangon Biotech).

**Table 1:** Sequences of primers used for qRT-PCR

Gene	Forward sequence	Reverse sequence
*Il1b*	AGTGTGGATCCCAAGCAATACCCA	TGTCCTGACCACTGTTGTTTCCCA
*Il6*	CGGAGAGGAGACTTCACAGAGG	GCCATTGCACAACTCTTTTCTCA
*Tnf*	ACTCCAGGCGGTGCCTATGT	GTGAGGGTCTGGGCCATAGAA
*Sdf1*	AGAGCCACATCGCCAGAGC	CATCACTGCCACCCAGAAGACTG
*Icam1*	AATGCCAGCTCGGAGGATCAC	CAGCCGAGGACCATACAGCA
*Cxcl5*	TCATGAGAAGGCAATGCT	ACATTATGCCATACTACGAAGA
*Igf1*	TAAGGAGGCTGGAGATGTAT	CTCTACTTGCGTTCTTCA
GAPDH	ATCCACTTTAATTTCGGGTCAATGC	ATGCCAGTGAGCTTCCCGTTC

Target gene expression was calculated using the 2^−ΔΔCt^ method, and the expression level of GAPDH was used as an internal reference.

### TUNEL assay

After 2 days of treatment, apoptosis was analysed by TUNEL assay kit (Elabscience) in paraffin sections of lung tissues of the four groups. Three sections were selected for each mouse and stained using the TUNEL assay kit according to the manufacturer's protocol, and the apoptosis of the lung tissue was observed under a fluorescence microscope in three fields of view for each section.

#### Immunofluorescence assay

Lung tissue sections were fixed and incubated with 3% bovine serum albumin (BSA) for 30 min at room temperature. Subsequently, the sections were incubated with an anti-proliferating cell nuclear antigen (PCNA) (1:100) (Proteintech). After washing with PBS, incubate with secondary antibody at 37 °C for 1 h. DAPI staining (Elabscience) was performed, and images were detected using a fluorescence microscope (Nikon Corporation).

#### Statistical analysis

GraphPad Prism 8.0 was used for statistical analysis. The normality of all data was tested using the Shapiro–Wilks test and all continuous variables were normally distributed. The data were presented as mean ± standard deviation. The one-way ANOVA was used for comparisons among groups, and multiple comparisons were performed with Tukey post hoc test. *P* < 0.05 was considered statistically significant.

## Results

### Optimization of pFUS parameters

**Fig. 1:**
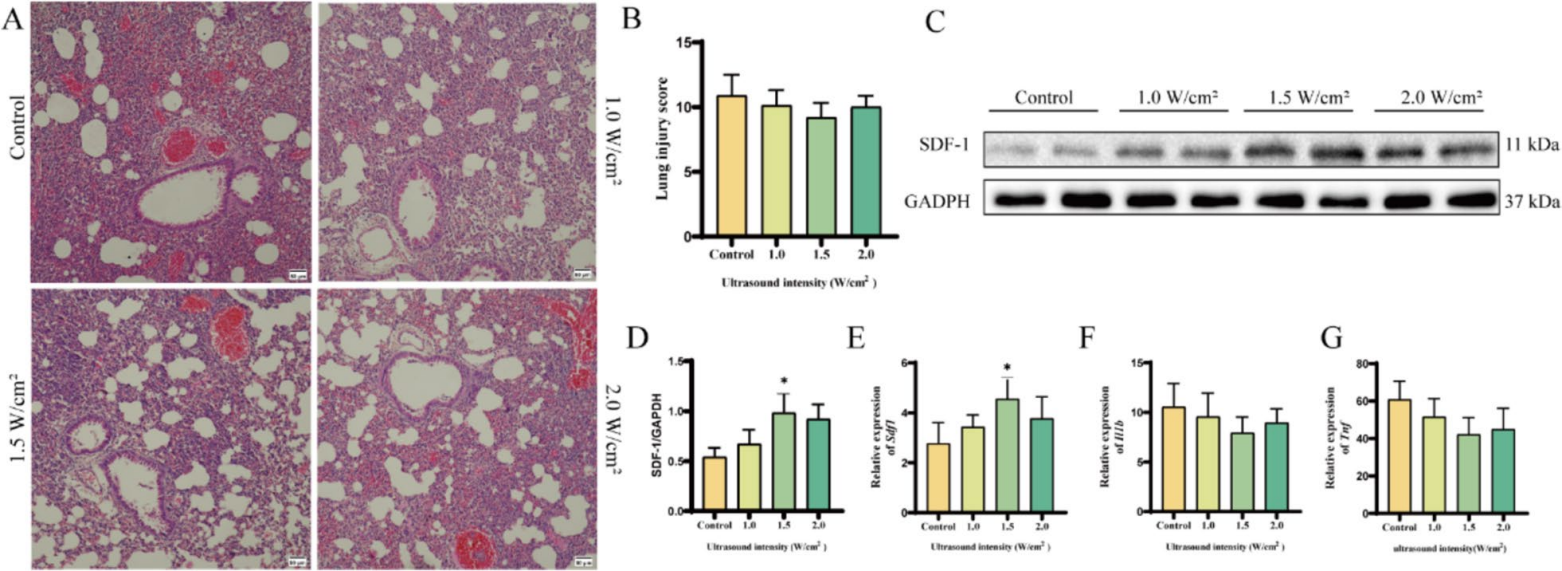
Effects of different pFUS intensities on ARDS mice (*n* = 4 per group). **A** Hematoxylin–eosin staining (HE staining, magnification × 200, scale bar:50 μm). **B** Lung injury scores were calculated according to the severity of lung injury. **C, D** Western blot analysis. **E, F, G** mRNA level of *Sdf-1*, *Tnf* and *Il1b* (*p < 0.05, vs. Control). Full-length blots/gels are presented in Supplementary file 2: Fig. S1

After receiving irradiation with different ultrasound intensities, HE staining of mouse lung tissues showed slight changes in alveolar wall damage in the 1.5 W/cm^2^ group, and the lung injury score showed a decrease in the 1.5 W/cm^2^ group, but was not statistically significant (Fig. 1A, B). The expression of SDF-1, an important factor of the migration ability, compared with the Control group, the protein expression levels of SDF-1 in the lung tissues of mice were elevated after pFUS intervention, and 1.5 W/cm^2^ was the most obvious increase, with a statistically significant difference (Fig. 1C, D). Detection of lung tissue mRNA by qRT-PCR was also consistent with Western blotting results, with the most pronounced elevation at 1.5 W/cm^2^ (1.0 W/cm^2^ vs. 1.5 W/cm^2^ vs. 2.0 W/cm^2^, 3.4 ± 0.5 vs. 4.6 ± 0.8 vs. 3.9 ± 1.0, Fig. 1E). The expression mRNA levels of *Tnf* and *Il1b* in lung tissue, both of which tended to decrease but were not statistically different, suggests that pFUS has no lung damaging effects and may even reduce lung inflammation. Therefore, 1.5 W/cm^2^ pFUS was finally chosen as the ultrasound parameter for this experiment (Fig. 1E, F).

### Effects of pFUS alone

**Fig. 2:**
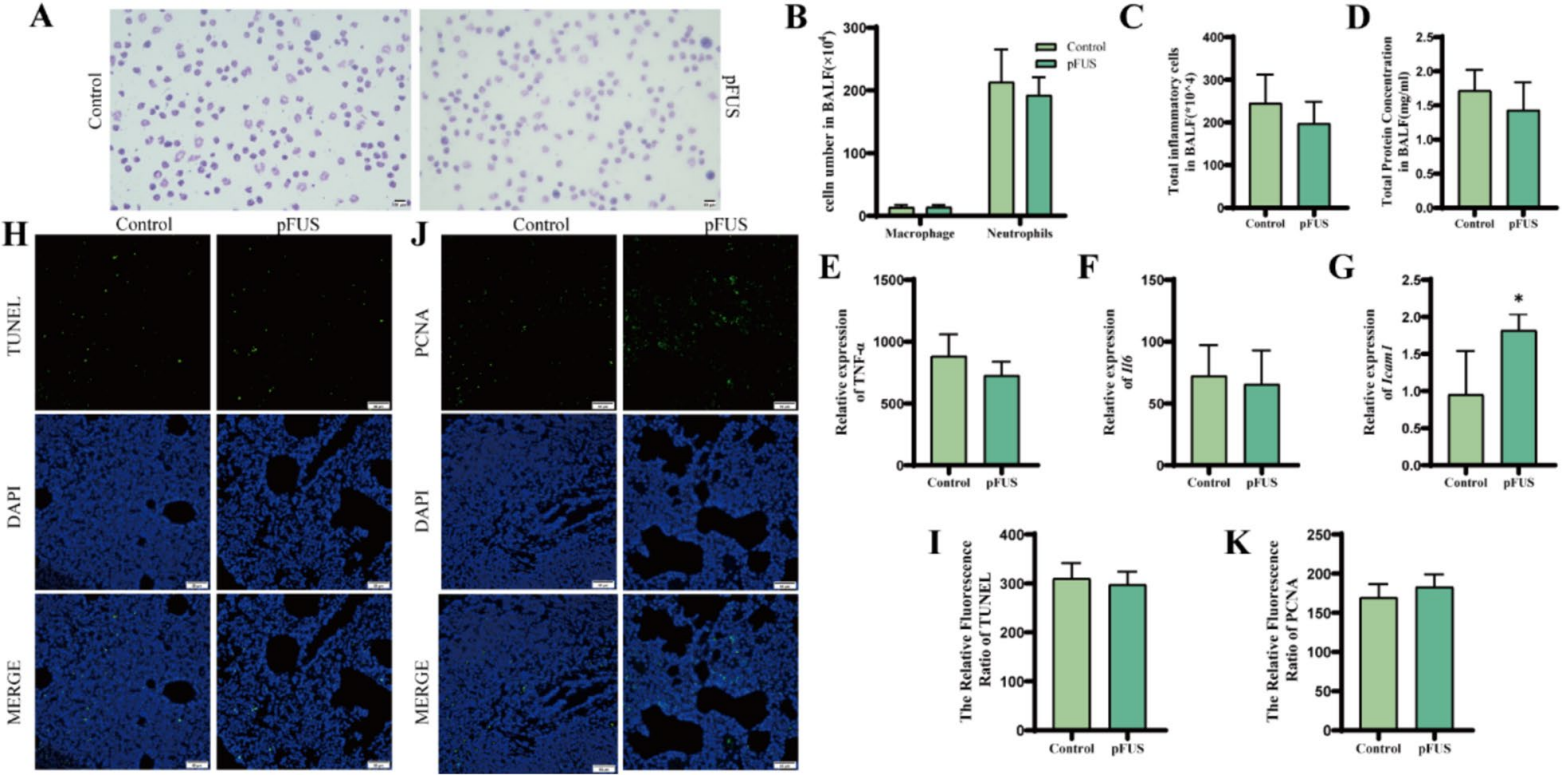
pFUS alone (no MSCs) does not alter ARDS outcomes (*n* = 4 per group). **A** Swiss Giemsa stained (magnification × 200, scale bar:50 μm). **B** Macrophage and neutrophil counts in BALF.** C** BALF total inflammatory cell count. **D** Total BALF protein concentration.** E** TNF-α levels in BALF. **F, G** mRNA level of *Il6* and *Icam1*. **H, I** Representative images of TUNEL staining. Scale bar: 50 μm. **J, K** Representative images of PCNA immunofluorescence staining. Scale bar: 50 μm

Since the effects of pFUS alone during ARDS were unknown, we examined whether using pFUS alone at a frequency of 1.5 W/cm^2^ altered ARDS progression. Compared with control mice, mice in the pFUS-only group showed a decrease in the number of BALF neutrophils, the number of total inflammatory cells, the concentration of total protein and TNF-α, the mRNA level of Il6 in lung tissue, and the apoptotic rate of lung tissue, and an increase in the number of BALF macrophages and the proliferation rate, all of which were not statistically different., however, lung tissue mRNA levels of *Icam1* appeared increased (Fig. 2 A-K).

### pFUS combined with hUC-MSCs to alleviate lung inflammation and lung injury

**Fig. 3:**
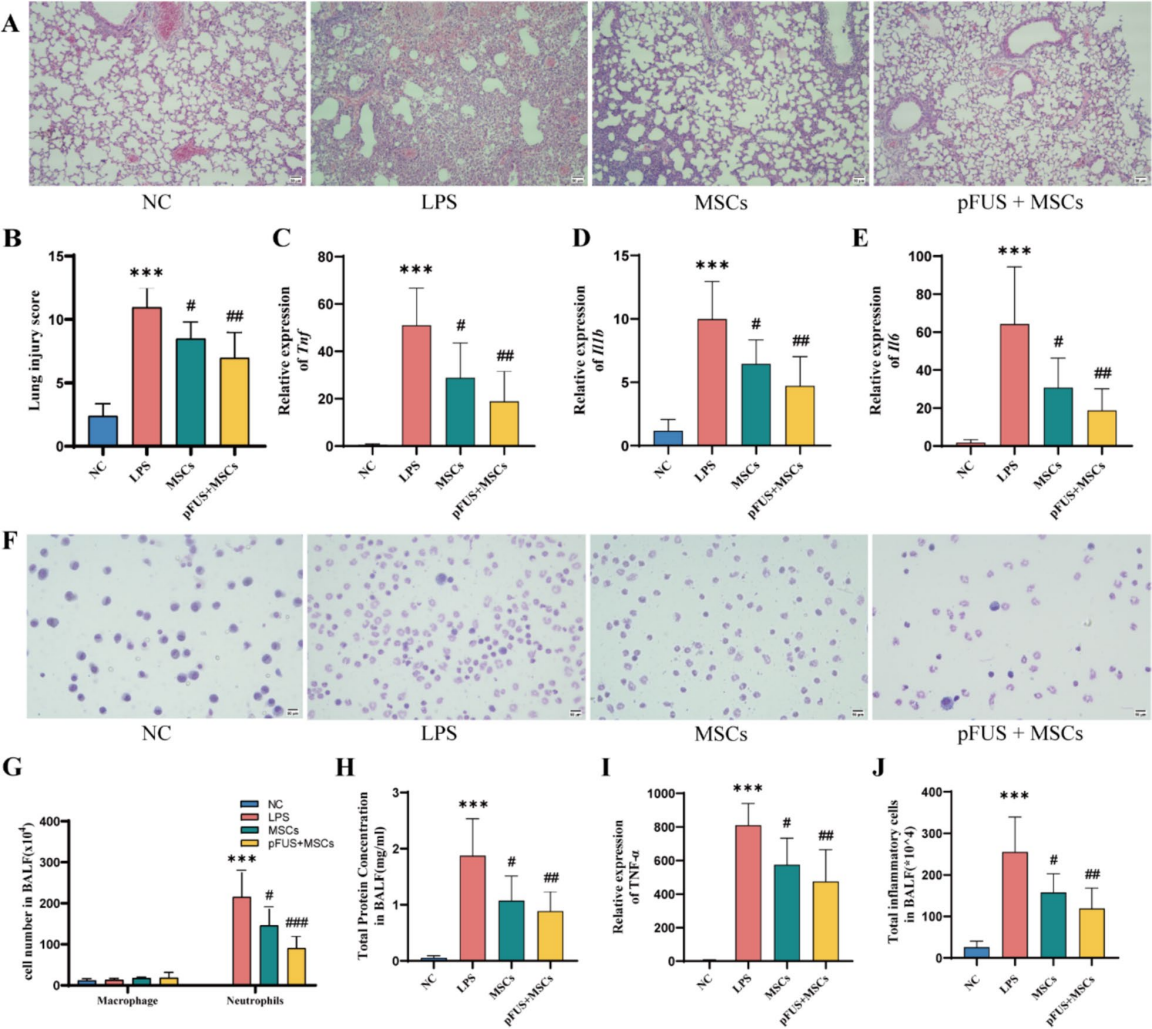
pFUS combined with hUC-MSCs in ARDS mice (*n* = 6 per group). **A** Hematoxylin–eosin staining (HE staining, magnification × 200, scale bar:50 μm). **B** Lung injury scores were calculated according to the severity of lung injury. **C, D, E** mRNA level of *Tnf*, *Il1b* and *Il6*. **F** Swiss Giemsa stained (magnification × 200, scale bar:50 μm). **G** Macrophage and neutrophil counts in BALF. **H** Total BALF protein concentration. **I** TNF-α levels in BALF. **J** BALF total inflammatory cell count. (*** P < 0. 001 vs. NC, #*P* < 0.05, ##*P* < 0.01, ###*P* < 0.001 vs. LPS)

The culture and characterisation of hUC-MSCs has been placed in Supplementary Fig S2. Compared with the NC group, mice in the LPS group had inflammatory cell infiltration in the lung tissue, structural damage, thickening of alveolar septa, large amounts of inflammatory cells and erythrocytes in the alveolar lumen, and increased lung injury scores, which were all improved after hUC-MSCs treatment. Treatment with hUC-MSCs improved these conditions, and compared to the hUC-MSCs treatment group, the pFUS + MSCs group had lower lung injury scores and more significant remission of lung injury and inflammatory infiltrates (Fig. 3A, B). Additionally, inflammatory markers *Tnf*, *Il1b and Il6* were higher in the LPS group but decreased significantly with hUC-MSCs and even more with pFUS + MSCs treatment (Fig. 3C-E). BALF analysis revealed that macrophages predominated in the NC group, while neutrophils were more common in the LPS group. Neutrophil levels decreased significantly with MSCs and pFUS + MSCs treatments, while macrophage numbers remained stable across groups (Fig. 3F, G). In the LPS group of mice, total BALF protein (Fig. 3H), TNF-α (Fig. 3I), and inflammatory cell count (Fig. 3J) were higher than in the NC group. These levels decreased when treated with hUC-MSCs or hUC-MSCs combined with pFUS, with the most significant reduction observed in the pFUS + MSCs group.

### pFUS with hUC-MSCs reduces apoptosis and boosts lung cell proliferation in ARDS mice

**Fig. 4:**
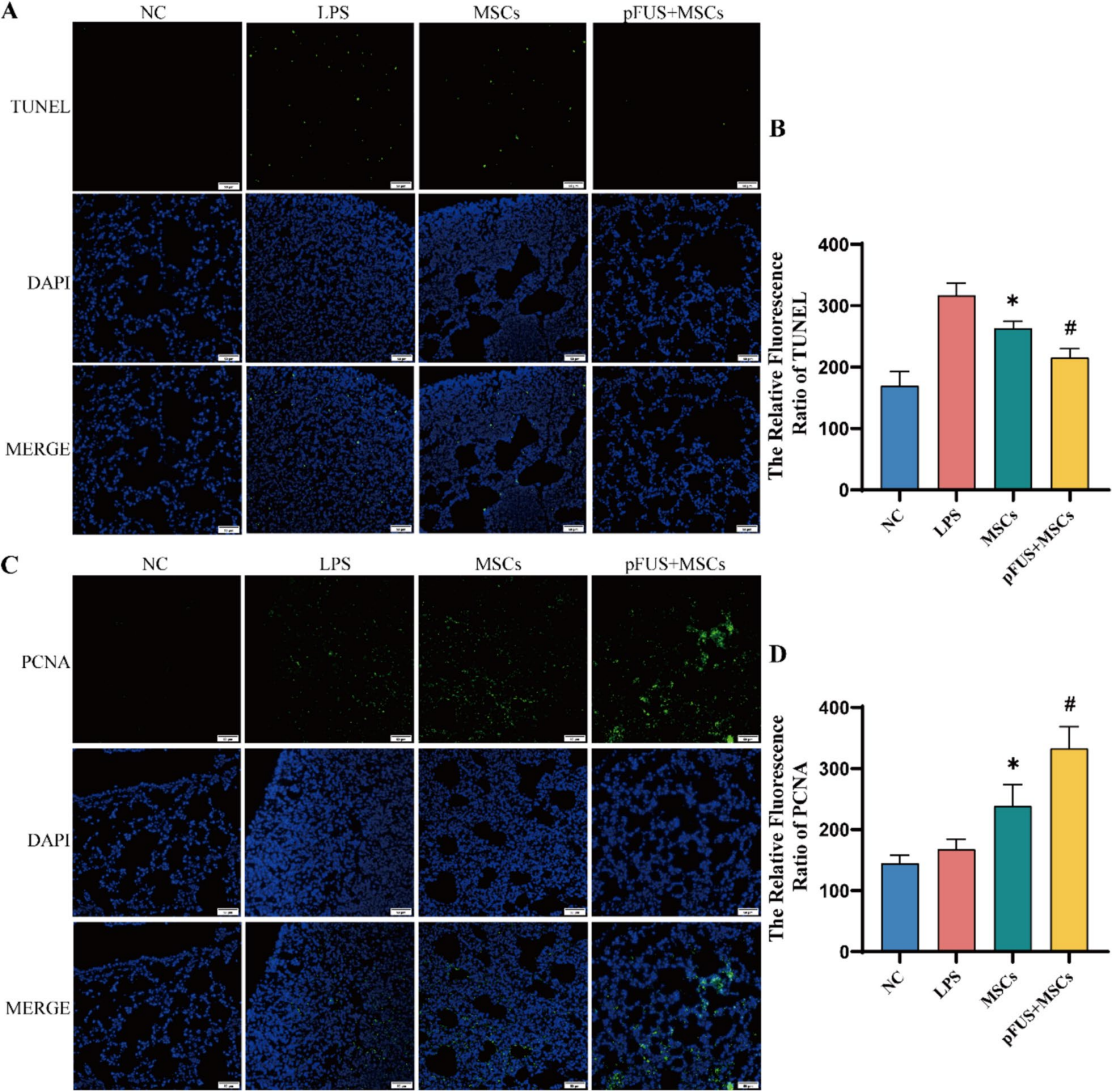
pFUS combined with hUC-MSCs attenuates lung apoptosis and promotes proliferation (*n* = 3 per group). **A, B** Representative images of TUNEL staining. Scale bar: 50 μm. **C, D** Representative images of PCNA immunofluorescence staining. Scale bar: 50 μm

TUNEL staining showed higher lung apoptosis in the LPS group compared to the NC group, while the MSCs and pFUS + MSCs groups had significantly lower apoptosis, with the pFUS + MSCs group showing the greatest reduction (Fig. 4A, B). PCNA staining indicated increased lung cell proliferation in the MSCs and pFUS + MSCs groups compared to the LPS group, with the pFUS + MSCs group exhibiting the highest proliferation (Fig. 4C, D).

### Up-regulation of homing-associated factors by pFUS promotes lung homing and survival of hUC-MSCs

**Fig. 5:**
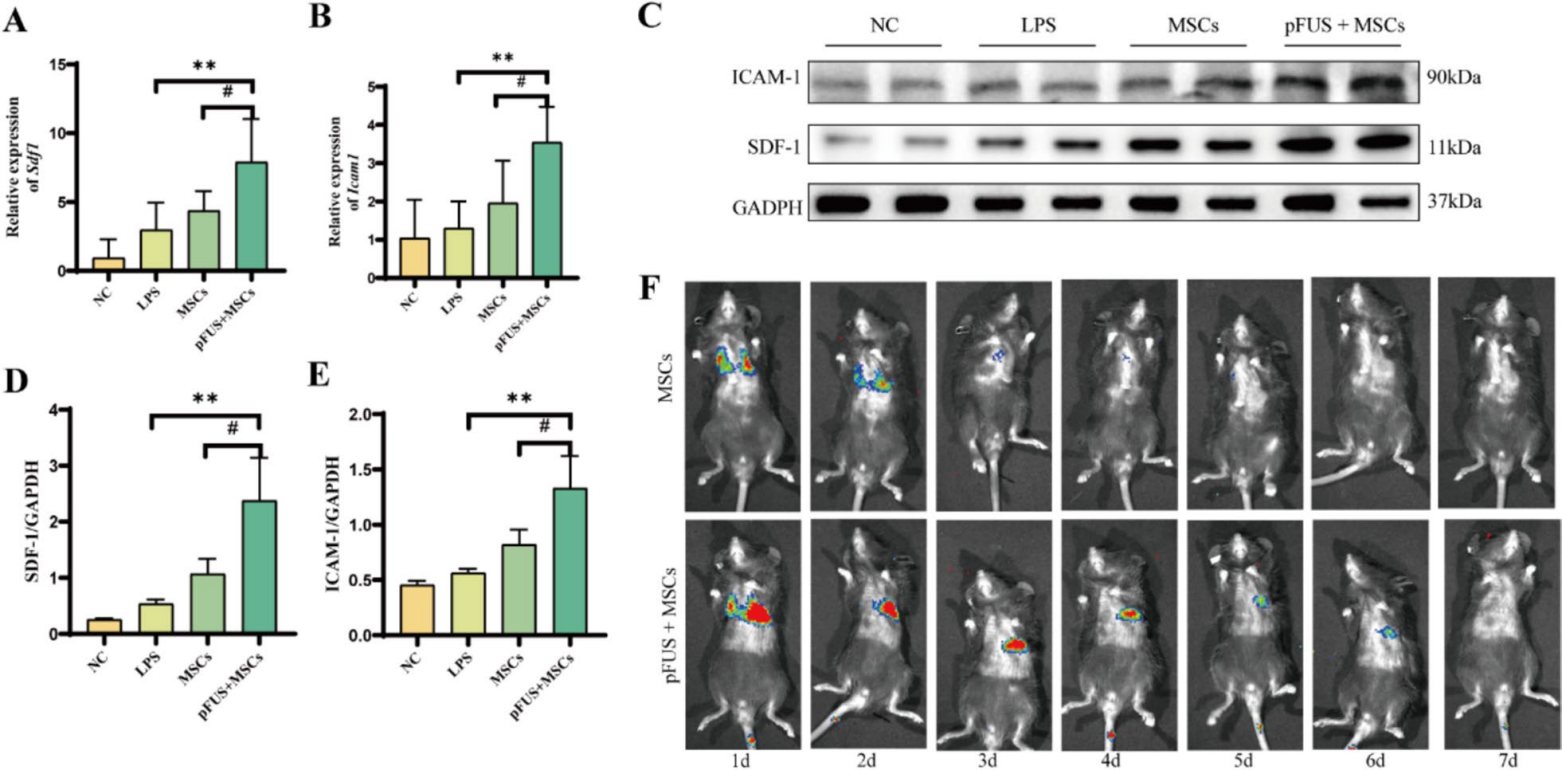
Homing and survival of hUC-MSCs in mouse lungs. **A, B** mRNA levels of *Sdf1*, *Icam1*(*n* = 6 per group). **C, D, E** Western blot analysis of SDF-1, ICAM-1 (*n* = 3 per group). **F** Bioluminescence imaging of mice one week after injection of hUC-MSCs (*n* = 3 per group). (**p < 0.01 vs. LPS, #*P* < 0.05 vs. MSCs). Full-length blots/gels are presented in Supplementary file 2: Fig. S2

Lentivirus-damaged hUC-MSCs showed bright green fluorescence under fluorescence microscopy (Supplementary Fig.S4). SDF-1 and ICAM-1 were upregulated after hUC-MSCs injection compared to the LPS group, and SDF-1 and ICAM-1 were significantly higher in the pFUS + MSCs group compared to the MSCs group (Fig. 5A-E). Bioluminescence imaging revealed that hUC-MSCs in the pFUS + MSCs group had a significantly increased homing rate relative to the MSCs group and prolonged survival in mouse lungs up to day 6 (Fig. 5F).

### Analysis of the mechanism by which pFUS promotes the homing of hUC-MSCs

**Fig. 6:**
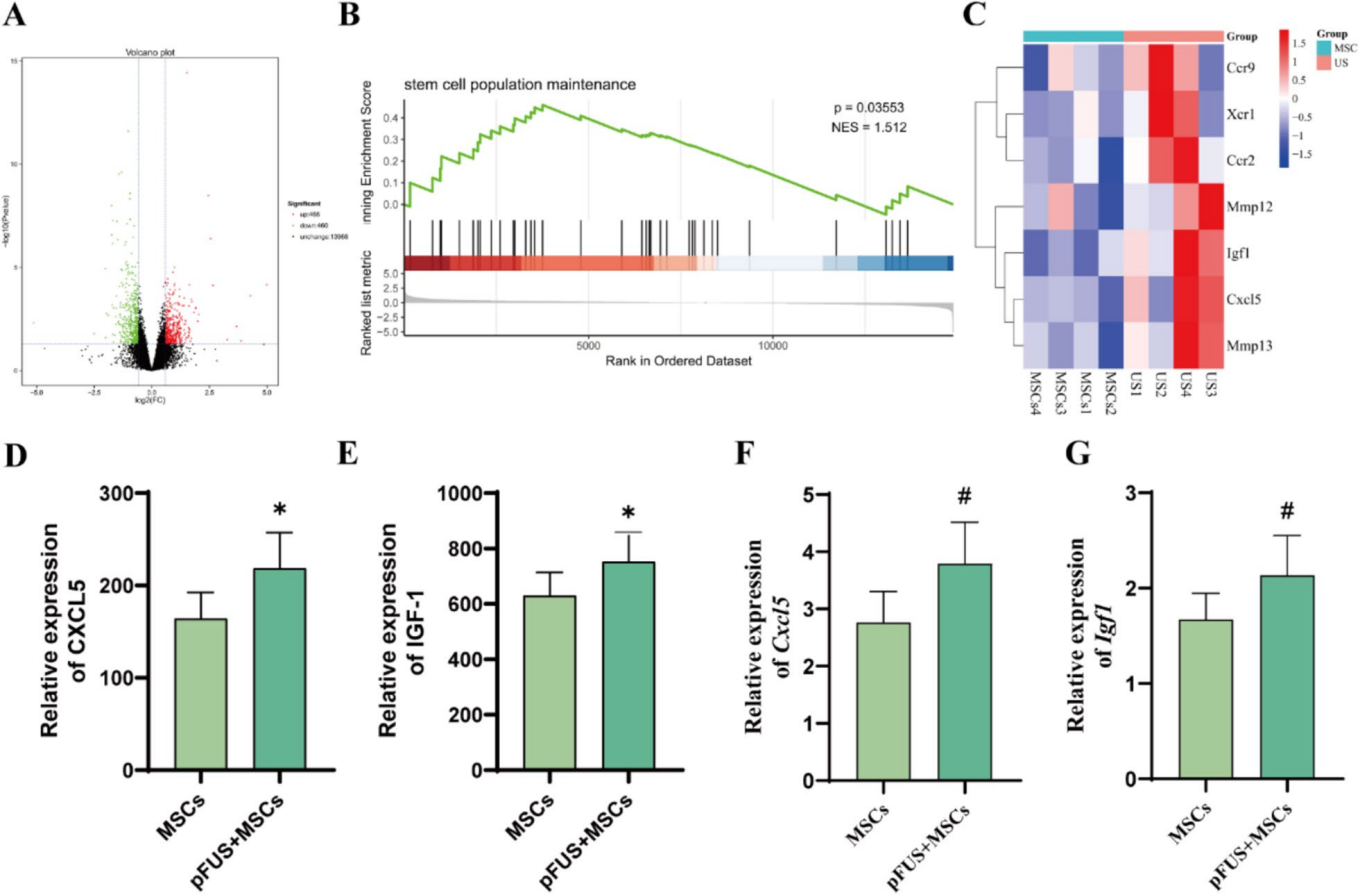
**G** Analysis of the mechanism by which pFUS promotes the homing of hUC-MSCs. **A** Volcano plots of DEGs in the MSCs group vs pFUS + MSCs group. Red dots indicate higher DEGs in the pFUS + MSCs groups compared to the MSCs group, and green dots indicate lower DEGs in the pFUS + MSCs group (n = 4 per group). **B** GSEA plots of DEGs in both MSCs group vs pFUS + MSCs group (GO:0019827, X-axis: ranking of all genes. NES, normalized enrichment score). **C** Cluster analysis of up-regulated DEGs of MSCs homing-related, each column represents one sample (US = pFUS + MSCs group). **D**, **E** mRNA levels of *Cxcl5* and* Igf1* in lung tissue (*n* = 3 per group). **F, G** CXCL5 and IGF-1 protein levels in BALF (*n* = 3 per group). (*P < 0.05 vs. MSCs)

To determine the effect of pFUS on the homing mechanism of hUC-MSCs, we performed transcriptomic analysis of mouse lung tissues. There were 466 up-regulated differentially expressed genes (DEGs) and 460 down-regulated DEGs in the pFUS + MSCs group compared to the MSCs group (Fig. 6A). We found an upregulation of the stem cell population maintenance process in the pFUS + MSCs group by GSEA(Fig. 6B).Furthermore, we screened up-regulated hUC-MSCs homing-associated factor genes between the MSCs and pFUS + MSCs groups, heat-map analysis showed that CC chemokine receptor 2 (*Ccr2*), *Ccr9*, X-C chemokine receptor 1 (*Xcr1*), *Mmp12* of the Matrix metalloproteinases (MMPs) family, *Mmp13*, insulin-like growth factor 1 (*Igf1*) and CXC motif chemokine ligand 5 (*Cxcl5*) were elevated in the pFUS + MSCs group (Fig. 6C). Among them, *Igf1* and *Cxcl5* may be pFUS-promoted MSC homing-promoting cytokines elevated in the lung injury setting, therefore, validation using qRT-PCR and ELISA showed that CXCL5 and IGF-1 appeared elevated in lung tissue and BALF in the pFUS + MSCs group compared to the MSCs group (Fig. 6D-G).

## Discussion

Our findings demonstrate that hUC-MSCs exhibit significant therapeutic potential in ameliorating LPS-induced ARDS, and pFUS irradiation of lungs increased chemokine levels, which led to more hUC-MSCs homing to target tissues and prolonged their retention time, which improved lung oedema, reduced lung inflammation, and promoted lung cell proliferation and reduced cell apoptosis.

Previous studies have reported that hUC-MSCs transplantation is both safe and feasible for the treatment of lung injury. Currently, hUC-MSCs transplantation is being utilized as an exogenous source of stem cells to repair injured lungs, particularly in conditions such as ALI/ARDS [17, 18]. The hUC-MSCs are more suited for treating lung diseases than bone marrow and adipose-derived MSCs, because they are richer in CD146, a critical cell adhesion protein in blood vessels and endothelium, and exhibit superior functional characteristics, such as enhanced adhesion and proliferation rates, additionally, hUC-MSCs possess several notable advantages, including high activity, easy accessibility, rapid proliferation, and low immunogenicity, and have been increasingly reported for their therapeutic potential in the treatment of various diseases [19–21]. Clinical study have shown that hUC-MSCs can effectively treat critically ill COVID-19 patients and exert positive effects on their long-term sequelae [22]. It has also been demonstrated that fecal microbiota transplantation in mice treated with hUC-MSCs can significantly mitigate acute lung injury [23]. Our research demonstrated that the administration of hUC-MSCs in a mice model of ARDS yielded significant therapeutic benefits, specifically, hUC-MSC treatment led to a marked reduction in alveolar septal thickening, a decrease in pulmonary hemorrhage, and a diminished infiltration of inflammatory cells. Furthermore, it downregulated the levels of inflammatory factors in BALF and lung tissue, curbed lung apoptosis, and enhanced lung cell proliferation. These findings indicate that hUC-MSCs exert a multifaceted therapeutic impact in ARDS.

However, a plethora of studies has indicated that, by intravenous injection of hUC-MSCs, their homing efficiency in the lungs is relatively low, and the retention time was also quite short, which significantly restricts their overall therapeutic efficacy [24, 25]. Previous studies in animal models of muscle injury, limb ischemia and kidney injury have found that pFUS up-regulates the local gradient of factors such as chemokines, growth factors and anti-inflammatory cytokines in the tissue microenvironment, specifically, all of which are involved in the activation, adhesion, and migration steps that guide MSCs to their target sites, and even an increase in the retention of MSCs in the kidneys [11, 26–28]. In addition, it was found that pFUS promotes the regulation of neural tissues through relevant mechanosensitive ion channels, and that it can target stimulation of the hepatic portal plexus to lower blood glucose concentrations in patients [29]. It has also been found that pFUS splenic irradiation alone alters inflammatory cytokine levels in models of acute endotoxaemia and pneumonia by modulating cholinergic anti-inflammatory pathways [30]. However, current studies have revealed that the ultrasound parameters selected for pFUS treatment vary widely across different investigations and there is still a lack of relevant studies in lung injury models, so in this study wwe employed a mouse model of ARDS induced by LPS to investigate the potential role and underlying mechanisms of optimized pFUS in promoting the homing of hUC-MSCs and enhancing ARDS treatment. To elucidate the biological effects of pFUS on the target lungs, the results of bioluminescence imaging showed that pFUS increased the lung homing rate of MSCs and significantly prolonged their survival time in the lungs. Furthermore, through molecular-level assays, we meticulously examined the expression profiles of two key chemotactic molecules, SDF-1 and ICAM-1, which are implicated in the homing of hUC-MSCs to the lungs [31–33]. Both western blotting and qRT-PCR showed that the expression of SDF-1 and ICAM-1 were significantly elevated in the pFUS + MSCs group, and the detected mRNA levels correlated well with the corresponding protein levels. The results revealed that upregulation of SDF-1 levels following pFUS pretreatment at 1.5 W/cm^2^ is associated with enhanced homing efficiency of hUC-MSCs into the lungs. Additionally, the increased expression of ICAM-1 indicated that pFUS may have augmented the permeability of intercellular and pulmonary interstitial capillaries, promoting efficient migration of hUC-MSCs. Our results are consistent with previous researches demonstrating that enhances cell implantation efficacy when pFUS is administered prior to the injection of hUC-MSCs [12, 26].

To explore the mechanism and specific aspects of pFUS to promote lung homing in hUC-MSCs, we conducted transcriptome sequencing to identify DEG, and we found that several key factors such as *Cxcl5*, *Igf1*, *Ccr2*, *Ccr9*, *Xcr1*, *Mmp12*, and *Mmp13* were increased after pFUS intervention. Previous studies have established that CXCL5 and SDF-1 (CXCL12) are members of the chemokine family, sharing analogous functions and playing a crucial role in the recruitment and migration of cells [34, 35]. Animal studies have demonstrated that MSCs engineered to overexpress IGF-1 can enhance the homing of additional MSCs to the infarcted myocardium, which is mediated through the paracrine release of SDF-1, and subsequently promotes the healing and repair of the damaged cardiac tissue [36]. Receptors such as CCR2 and CCR9, which are secreted by MSCs, along with SDF-1 secreted by endothelial cells at the site of injury and CXCL5 secreted by type II alveolar epithelial cells in lung injury, are all implicated in the second step of cell activation during the homing process, secondly, MSCs may secrete MMP to facilitate in the fourth step of homing in which MSCs penetrate the endothelial cell layer and basement membrane for migration or detachment, meanwhile, macrophages and endothelial cells at the site of injury may secrete IGF-1 to play a crucial role in the fifth step of homing in which MSCs migrate through the stroma to the damaged site [7, 37–39]. In addition, we confirmed that the levels of CXCL5 and IGF-1 were significantly higher in the pFUS + MSCs group than in the MSCs group, as verified by ELISA and qRT-PCR assays. The results suggest that pFUS may enhance the homing efficiency hUC-MSCs and prolong their maintenance within the lung tissue by upregulating IGF-1 and CXCL5 in the damaged lung microenvironment. In conclusion, pFUS may promote hUC-MSCs homing by modulating the expression of corresponding molecules, thereby facilitating the second step of activation and the fifth step of migration.

Our study has several limitations, we did not fully elucidate in depth the specific mechanism by which pFUS promotes the homing of hUC-MSCs and its subsequent therapeutic mechanisms. Currently, MSCs have been clinically utilized in the treatment of ARDS patients, however, we have not yet conducted clinical trials to verify the efficacy of combining pFUS with hUC-MSCs. This gap will be addressed in our future studies. Additionally, plan we to further explore related questions, such as whether pFUS application might influence the fate of hUC-MSCs following transplantation.

## Conclusion

In the present study, pFUS appears to have facilitated the activation and migration steps in the homing of hUC-MSCs by modulating homing-associated factors SDF-1, ICAM-1, CXCL5 and IGF-1, thereby promoting their lung homing, which in turn induced significant histological and cytokine improvements in ARDS. We provide a preliminary proof-of-concept that pFUS binding to transplanted hUC-MSCs is feasible and effective, showing therapeutic efficacy in treating a lung model of ARDS in mice.

## Supplementary Information

Additional file 1.

## Data Availability

The data sets analyzed during this study are available from the corresponding author on reasonable request. RNA sequencing data reported in this study have been submitted to the NCBI Gene Expression Omnibus database (https://www.ncbi.nlm.nih.gov/geo/) under accession number PRJNA1228302.

## Abbreviations

hUC-MSCs: Human umbilical cord mesenchymal stem cells
ARDS: Acute respiratory distress syndrome
pFUS: Pulsed focused ultrasound
EGFP: Enhanced green fluorescent protein
qRT-PCR: Quantitative reverse transcription-Polymerase Chain Reaction
HE: Hematoxylin and Eosin staining
TUNEL: Terminal deoxynucleotidyl-transferase-mediated dUTP nick end labeling
CD: Cluster of differentiation
DAPI: 2-(4-Amidinophenyl)-6-indolecarbamidine
PBS: Phosphate buffer saline
TBST: Tris-buffered saline with Tween20
