# Supplementary material for: Ultrasound assisted homing of human umbilical cord mesenchymal stem cells promotes recovery from acute respiratory distress syndrome
Source: Stem Cell Res Ther. 2025 Jul 26;16:407. doi: 10.1186/s13287-025-04545-6 (PMC12296617; doi:10.1186/s13287-025-04545-6)
Supplement: Supplementary file 1 — Additional file 1. [file 13287_2025_4545_MOESM1_ESM.docx]

Supplementary Figure S1


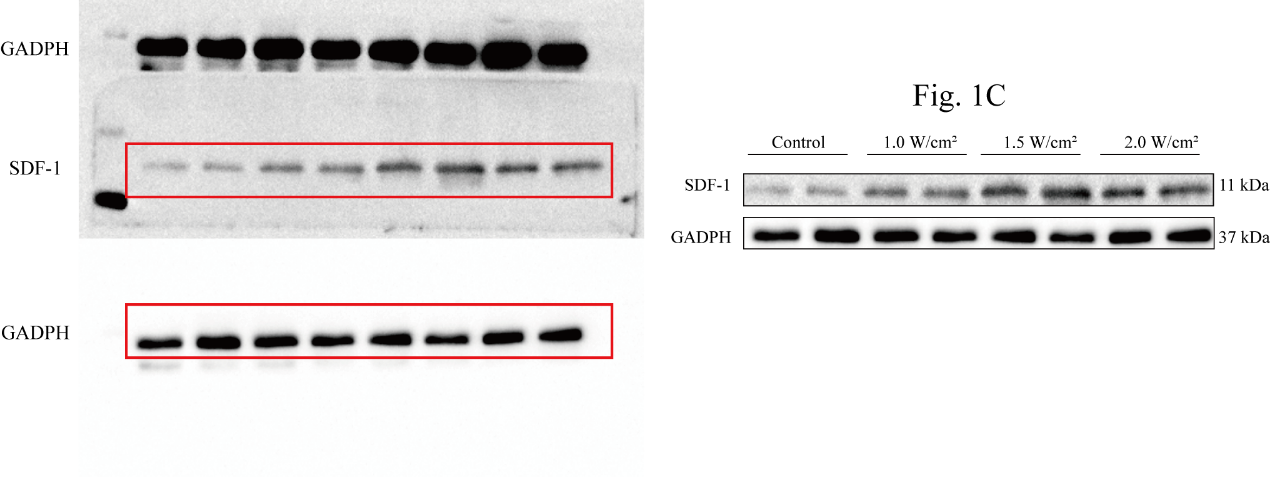


Uncropped immunoblots for Figure 1C

Supplementary Figure S2


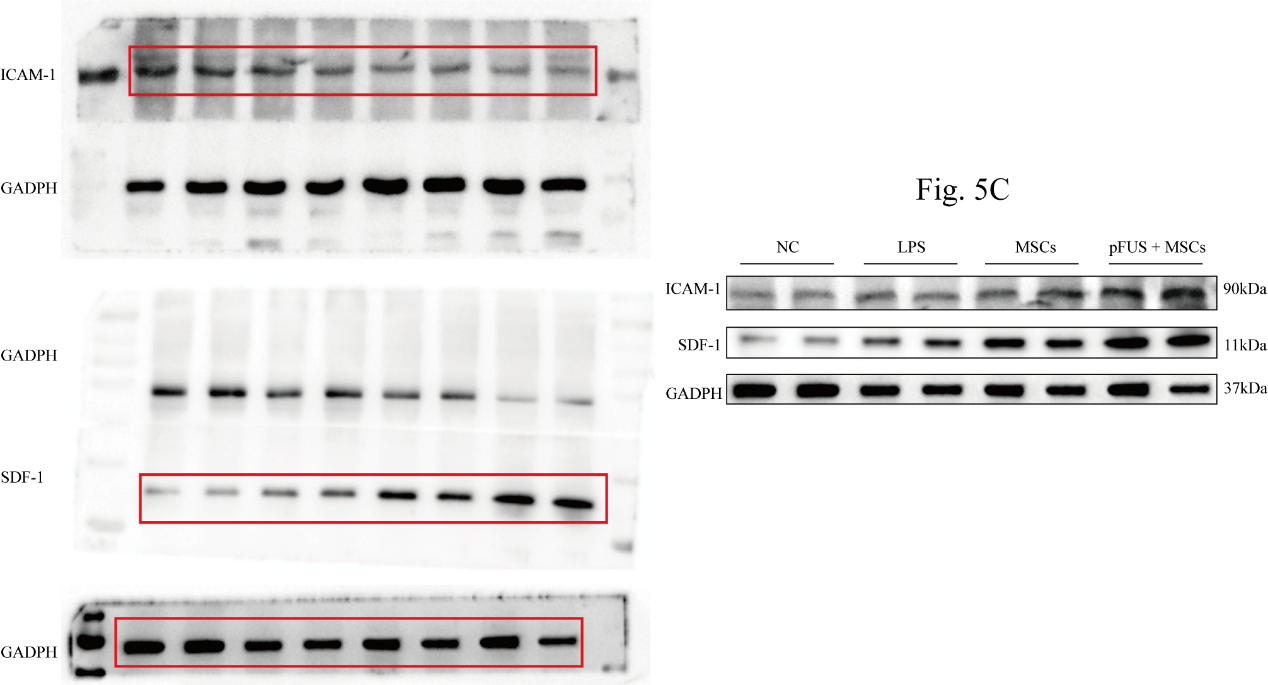


Uncropped immunoblots for Figure 5C
